# Case report: A rare presentation of high risk epithelioid hemangioendothelioma on leg treated with surgical excision

**DOI:** 10.1016/j.ijscr.2019.05.010

**Published:** 2019-05-10

**Authors:** Joshua Kazdan, Victoria Sharp, John Pui

**Affiliations:** Beaumont Farmington Hills Hospital, Farmington Hills, MI, 48336, USA

**Keywords:** Vascular tumor, Epithelial hemangioendothelioma, High risk, Invasive, Epithelioid, Pathology, Surgical excision

## Abstract

**Introduction:**

A rare vascular tumor, epithelioid hemangioendothelioma (EHE), can be difficult to diagnose for physicians. Although uncommon, EHE has the potential to become malignant and cause patient death. The five year mortality rate with the diagnosis of the high risk type has been reported to be as high as 41. Thus this finding requires aggressive treatment to prevent amputation or death (Deyrup et al., 2008).

**Presentation of case:**

In this case, a 60-year-old male patient was evaluated for a suspicious subepidermal nodule on the upper lateral aspect of the lower right leg just distal to the knee. It was excised to the level of muscle with appropriate margins of 1 cm circumferentially. The specimen underwent appropriate pathological testing and did reveal a high-risk-type epithelioid hemangioendothelioma with remaining tumor present at the deep margin. The patient had additional surgery by an orthopedic surgeon in order to obtain clear margins of the lesion and retain maximum function of leg. Despite surgical excision with ultimately clean margins, the lesion did metastasize to the right groin area seven months after surgery and the secondary metastatic site also required surgical excision.

**Discussion:**

There is very little information available to the identification and treatment of a finding of EHE. The only definitive treatments to prevent malignancy is excision or amputation, possibly in conjunction with radiation therapy. The role of oncology intervention should be considered because the finding may be either a cause or an effect of malignancy.

**Conclusion:**

The goal of this paper is to raise awareness of the importance of pathology for soft issue lesions even if there is initially a low clinical index of suspicion. Unique characteristics in the pathology is the cornerstone to the identification and treatment. Although surgical excision can appear to be a curative treatment, EHE may inevitably metastasize, so aggressive and definitive treatment is best.

## Highlights

• High risk type hemangioendothelioma (EHE) identified via pathology with characteristic nuclear pleomorphism and associated erythrocytes within vascular channels.
• CD31 membrane positivity with >3 mitosis/50 on high power field. Cytologic features of such a finding.
• Treated with surgical excision on leg (x2) with resolution of local site. Secondary site of EHE discovered at 7 month mark right groin mass which was also excised.
• Identified how to approach and treat a rare vascular tumor (EHE) type based upon limited literature review.

## 1 Introduction/background

Hemangioendotheliomas are considered to be tumors of intermediate malignancy because of their propensity to recur and occasionally metastasize, but at a rate lower than that of classical angiosarcomas [2]. There are several subtypes of hemangioendotheliomas; kaposiform hemangioendothelioma, papillary intralymphatic angioendothelioma, retiform pseudomyogenic hemangioendotheioma, and EHE [3]. The prevalence of epithelioid hemangioendotheliomas is extremely rare, accounting for less than one in a million in the general population as reported in the literature [3]. The tumor develops from connective tissue of pre-endothelial or vascular endothelial origin and can be found in many body locations, including the lungs, liver, heart, bone, central nervous system, and various other body locations as well, in multiple reports [4]. EHE is most commonly found in soft tissues. Lesions presenting in this fashion can represent either a primary lesion or a secondary site of metastasis. Due to the limited literature on the subject, it is not clear which age group is most affected, but it seems to appear in young to middle aged individuals more often [[1], [2], [3]]. There have been reported cases of EHE, to our knowledge, from age 9–93 [1]. It seems to have slight predominance in females [4]. EHE has been described as exhibiting sarcoma-like behavior at times and although the majority of cases do not result in mortality, it does have the ability to metastasize and cause death to patients [1]. Following a brief review of the literature, a case report will be discussed in this article of a rare high risk type finding of an EHE. The work has been reported in line with the SCARE criteria [5]

According to the World Health Organization, EHE is considered intermediate malignancy. In an article by Deyrup, 49 cases of EHE were examined to determine the risk stratification between low and high risk type of EHE. The study showed that when the tumor had increased size >3.0 cm or mitosis >3 mitosis/50 high power fields (hpf), it was associated with a worse prognosis and should be considered high risk type. The high risk type was associated with 59% 5 year survival while low risk did not have any deaths in their study. Metastasis occur in 25% of cases and the 5 year mortality rate is 19% [1].

The uniqueness of EHE as a distinct vascular tumor is due to WWTR1 (protein known as TAZ)-CAMTA1 (WC) fusion oncoprotein. This immunohistochemical marker, TAZ-CAMTA 1 (TC), which is the result of a t(1;3)(p36.3;q25) translocation appears to be found in EHE in greater than 90% of cases [3,6,7]. This translocation is a consistent abnormality as shown by a retrospective analysis by Errani et al. In their study, they showed 17 cases of EHE to have this translocatoion, as confirmed by FISH and RT-PCR testing. The translocation was not found when compared with other skin lesions including epithelioid hemangiomas, epithelioid angiosarcomas, and epithelioid sarcoma like EHE [5]. The transcription factors drive the cancer to develop. Understanding the chimeric transcription factors may have important implications in future as a target for treatment therapeutically [7]

Clinically, the lesion may be difficult to distinguish as it can appear in several locations and can resemble many other soft tissue tumors. EHE is mostly identified by unique pathological characteristics and immunohistochemistry findings [3,9]. The degree of pleomorphism, shape of nuclear membranes, chromatin distribution, and presence of nuclei all need to be examined under high power microscopy to determine diagnosis and degree of risk. A malignant feature of tumor cell spindling is associated with a worse prognosis [1]. These cytologic features can be used to guide how aggressive treatment should be.

Treatment options for EHE vary depending on low or high risk type. As these cases are rare in nature, each treatment should be individualized. The basis of treatment, if possible, is wide excision of the tumor, followed by chemotherapy or radiation therapy in some cases. There have been several case studies showing some promising anti-angiogenesis agents, particularly for unresectable tumors. One such agent, pazopanib, may be a therapeutic option to help control unresectable tumors [8]. Combination therapy has been reported sparsely with one case showing 90% reduction in left pleural effusion with carboplatin, paclitaxel, and bevacizumab. Bevacizumab has been used as monotherapy or with additional agents as well to cause disease to be stable [3]. A variety of other pathways and genetic tailoring have been theorized and tested. Hepatic EHE has even been treated with liver transplant. If a suspicious lesion, excisional surgery with wide margins is the preferred treatment option. Early diagnosis is important to long term outcome of the patient. 5 year survival is reduced to an alarming 30% if found in unresectable advanced stage [8]. If any question exists as to whether the tumor has been removed in its entirety with appropriate margins, it would be prudent to have oncology determine if additional treatment would be warranted. In this report, we present a patient of an apparent primary skin lesion identified as EHE with no metastasis surgically excised in two subsequent surgeries.

## 2 Case report

A 60 year old Caucasian male patient was referred to general surgery for multiple unrelated complaints including umbilical hernia and left arm lipoma. The patient also had an additional complaint of a fast-growing right leg mass located on upper lateral right calf distal to the knee. The patient had no other suspicious skin lesions and admitted to having the lesion shave biopsied two years prior by dermatologist with benign findings. No picture was taken of the lesion prior to surgical intervention as it was expected to be benign based upon prior dermatological findings. The patient stated that the leg lesion was non-painful in nature but was concerned that it may have increased in size over the previous 2 months and had a brown-gray discoloration. No prior imaging was obtained for the leg lesion. A wide margin elliptical excision was performed with a minimum goal of 1 cm margins on all sides of the lesion and the depth was resected to the muscle layer. The full specimen was marked for orientation and submitted to pathology. The excised elliptical portion measured 4.3 cm in length and 2.5 cm in width at widest points. The nodular lesion measured 2.4 × 1.8 × 0.9 cm. Ancillary studies showed that the lesion was CD31 positive, CD34 positive, and negative for cytokeratin markers. The pathology report confirmed EHE with tumor close to circumferential margins and present at the deep margin. The lesion was staged as pT1a pNX in accordance with AJCC staging. Given the deep margin extending to the thin layer of muscle just distal to the knee, the patient was referred to orthopedic surgery for further evaluation and operative intervention. The mainstay of treatment for invasive sarcoma is surgery often coupled with radiation and/or chemotherapy. A second surgical excision 19 days later following the original surgery was performed into deeper tissue. The second lenticular ellipse measured 8.1 cm in length and 2.1 cm in width at widest points at a depth of 1.4 cm. Multiple frozen sections were examined and clean margins of a minimum of 1 cm was determined in all directions.

Due to the diagnosis of EHE, it was prudent to obtain additional imaging to determine if the malignancy had metastasized. Although incredibly rare, there have been documented cases of pulmonary epithelioid hemangioendotheliomas, as well as cases of tumors found on the liver. A CT scan was performed with IV and oral contrast of the chest and abdomen. The findings were unremarkable for the chest but multiple hepatic cysts and an enhancing lesion in the right lobe of the liver were identified. A follow up MRI with and without gadolinium confirmed a 2 cm well-defined focal area of delayed enhancement within the posterior segment of the right lobe of the liver corresponding to the CT findings, likely representing a cyst and not a metastatic lesion. These findings suggest that no metastasis had occurred and that the leg skin lesion appeared to be the primary site of EHE.

## 3 Pathology

**Fig. 1:**
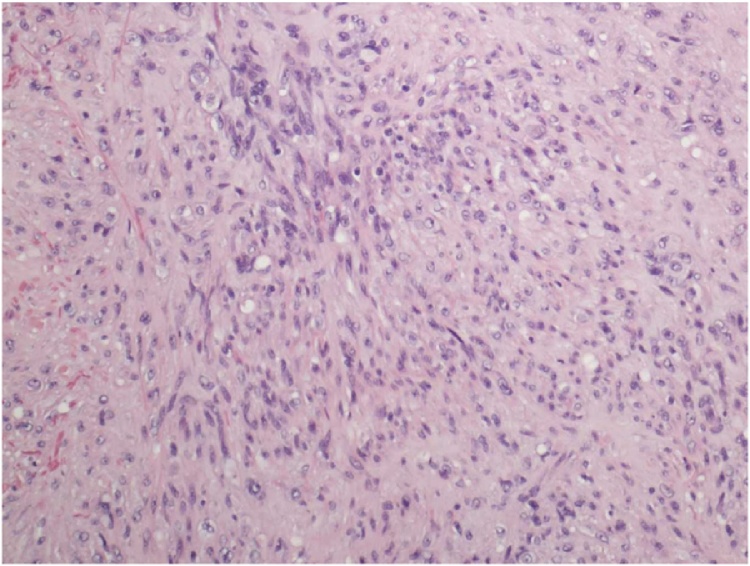
(H & E, original magnification 200×) Areas of both epithelioid and spindle cells within the tumor.

**Fig. 2:**
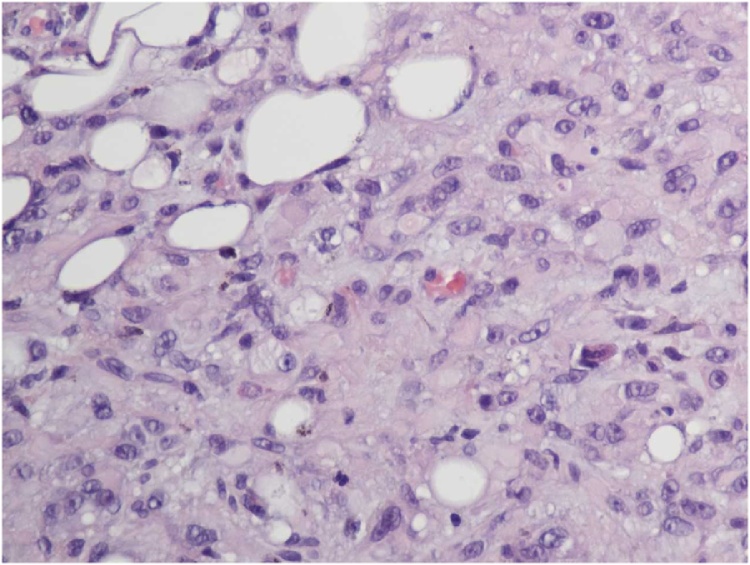
(H & E, original magnification 400×) Area of increased nuclear pleomorphism and associated erythrocytes within vascular channels.

**Fig. 3:**
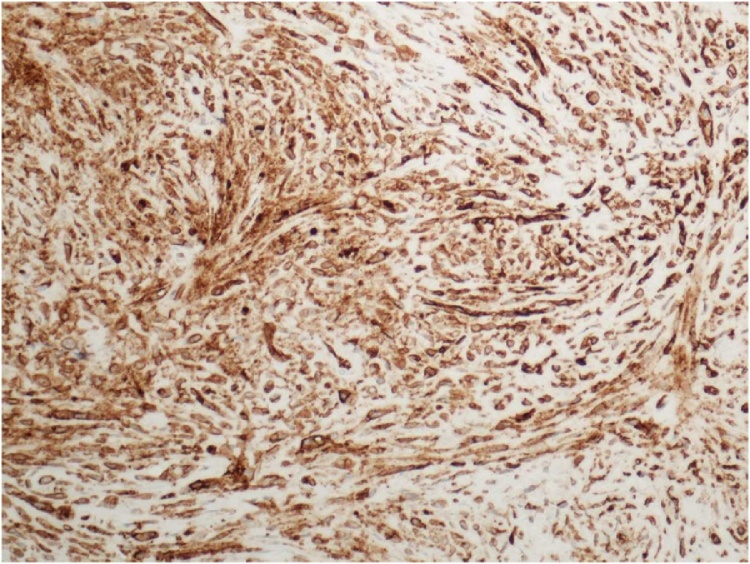
CD31 membrane positivity, confirming endothelial cell differentiation.

**Fig. 4:**
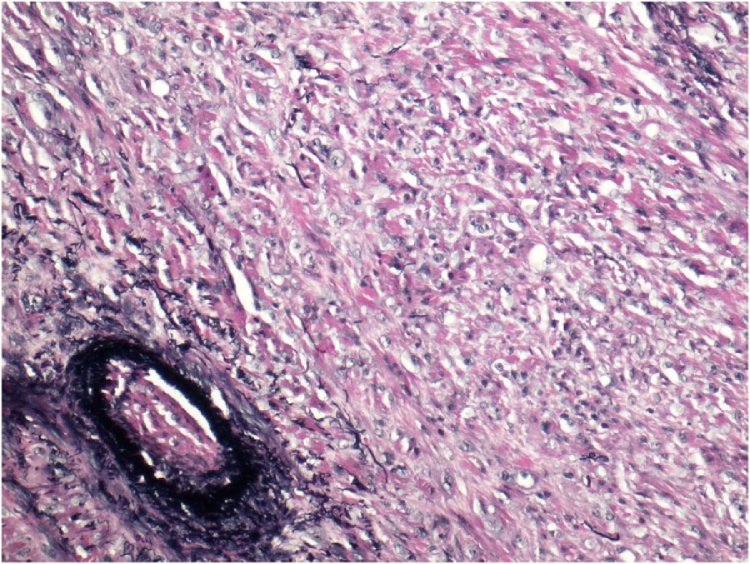
Elastic stain highlighting residual elastic intima and lamina of embedded vessel, possibly representing original site of origin.

The identification of the skin lesion as EHE was determined by a combination of microscopic, immunohistochemical, and gross pathologic findings. The gross pathology showed a raised brown-gray firm nodule measuring 2.4 × 1.8 × 0.9 cm in size within an ellipse of hair-bearing skin measuring 4.3 × 2.5 × 1.2 cm. Microscopic analysis showed a dermally centered proliferation of epithelioid to spindle shaped cells arranged in broad intersecting fascicles and lobules. Spindle shaped cells can be observed within the vascular tumor as observed in Fig. 1. Focal areas of hyalinization and necrosis were seen. The lesional cells had pleomorphic nuclei with prominent nucleoli and variable amounts of eosinophilic cytoplasm. In areas, cytoplasmic vacuoles were identified, and erythrocytes are seen in a few of these vacuoles. In Fig. 2, microscopic analysis shows erythrocytes which can be visualized in the vascular channels. The full immunohistochemistry panel showed that the lesional cells were positive for CD34, CD 31, ERG, CD10, muscle specific actin, INI1, and polytypic cytokeratin. The positive histochemistry of CD31 is shown in Fig. 3. The negative immunohistochemistry markers tested were CK 5/6,pP63, CK AE1/3, Mart-1, SOX10, S100, and desmin. Shown in Fig. 4 is the elastic stain highlighting residual elastic intima and lamina of embedded vessel, possibly representing original site of origin.

In the largest cytologic study of EHE to date by Murali et al., 11 histologically confirmed EHEs all showed occasional intranuclear pseudoinclusions and intracytoplasmic lumina found in epithelioid cells, whereas only 45% cases showed rare erythrocytes [10]. Rare mitotic figures were identified. The greatest risk factors according to a retrospective analysis performed by Deyrup et al. states the greatest risk factors are mitotic value and size. If either size or mitotic value is above the appropriate threshold, then it is classified as high risk [1]. The nodule was below the 3.0 cm threshold only measuring 2.4 cm in its greatest dimension. The mitotic value, however, in our case was 4 mitosis/50 hpf, which is above the >3 mitosis/50 hpf threshold, classifying the tumor as high risk. Since this lesion is of high risk type, it warranted aggressive treatment with ancillary imaging to rule out metastasis.

## 4 Follow-up

At 6 months follow up the patient had not had any recurrence in the area and was fully healed. The patient, however, had a right inguinal mass excised at 7 months that also was confirmed to be a metastatic site of epithelioid hemangioendothelioma. This metastatic lesion was surprising as the right growing mass had previously been tested as acellular. Even more surprising, flow cytometry was positive for CLL, which was not previously identified in this patient. The patient did have a follow up PET/CT scan which showed a 1.3 cm soft tissue nodule superior to the resection cavity. Patient was also referred to radiation oncology and will likely start radiation treatments six days a week. He will also require further lymph node resection due to new mass findings. This patients follow up is ongoing and will need continued multi-specialty care.

## 5 Conclusion

Due to the rarity of this dermatological finding, the literature is very limited. The importance of physicians being aware of the features of EHE allows more accurate identification of this vascular tumor. Treatment options continue to expand as individual case reports with various agents have been shown to be promising. Anecdotal evidence of pharmacologic agents will require larger population and long term studies to determine the effectiveness of anti-angiogenesis agents. EHE in soft tissue has the potential to be either a primary lesion or site of metastases. Patients should also have additional imaging if EHE is found on the skin to ensure that it is not a site of metastasis. Five year mortality rate is significantly increased in high risk findings based on microscopic appearance and should be treated in a more aggressive, multimodal manner. As shown in this case study, a second site of metastasis was identified because of a high index of suspicion and appropriate oncological surveillance. It is the hope of this article to raise interest in EHE and to spread awareness that although surgical resection can be an effective means of treatment. It may, however, not be curative and close patient monitoring is of the utmost important.

## Conflicts of interest

None.

## Funding

None.

## Ethical approval

Exempted by our institution since it is a single case report.

## Consent

Verbal and Written consent obtained.

## Author contribution

Joshua Kazdan-Writing of paper.

Victoria Sharpe-Primary surgeon.

John Pui-Images, pathology analysis.

## Guarantor

Joshua Kazdan.

## Provenance and peer review

Not commissioned, externally peer-reviewed.
